# Effects of several tea extracts on nonalcoholic fatty liver disease in mice fed with a high‐fat diet

**DOI:** 10.1002/fsn3.2255

**Published:** 2021-04-09

**Authors:** Qian‐Qian Mao, Bang‐Yan Li, Jin‐Ming Meng, Ren‐You Gan, Xiao‐Yu Xu, Ying‐Ying Gu, Xiao‐Hui Wang, Hua‐Bin Li

**Affiliations:** ^1^ Guangdong Provincial Key Laboratory of Food, Nutrition and Health Department of Nutrition School of Public Health Sun Yat‐Sen University Guangzhou China; ^2^ Research Center for Plants and Human Health Institute of Urban Agriculture Chinese Academy of Agricultural Sciences Chengdu China; ^3^ Key Laboratory of Coarse Cereal Processing (Ministry of Agriculture and Rural Affairs) Sichuan Engineering & Technology Research Center of Coarse Cereal Industrialization Chengdu University Chengdu China

**Keywords:** antioxidant, nonalcoholic fatty liver disease, obesity, prevention, tea

## Abstract

Nonalcoholic fatty liver disease (NAFLD) is considered as a severe threat to human health. It has been reported that tea has abundant bioactive compounds and beneficial effects. In our study, the effects of 12 tea extracts on NAFLD were assessed and compared at the dose of 200 mg/kg body weight in mice fed with a high‐fat diet (HFD) for 15 weeks. Enshi Yulu Tea, Fenghuang Narcissus Tea, and Yihong Tea showed strong effects in suppressing the accumulation of epididymal and perirenal adipose tissue as well as the increases of body weight and liver weight. The histopathological analysis revealed that hepatic steatosis and adipocyte hypertrophy induced by a HFD could be ameliorated by tea supplementation. In addition, Enshi Yulu Tea and Qing Brick Tea exerted more remarkable functions on decreasing the level of serum triglyceride and preventing hepatic fat accumulation, respectively. Furthermore, Fenghuang Narcissus Tea, Enshi Yulu Tea, and Qing Brick Tea could reverse the abnormal change in the levels of glutathione and superoxide dismutase. Moreover, 13 phytoconstituents were detected and quantified in these teas with high‐performance liquid chromatography (HPLC) method. The correlation analysis demonstrated that gallic acid might decrease MDA level, and the reduction of liver weight might be attributed to ellagic acid. However, it should be paid attention to some teas that showed hepatotoxicity with elevated levels of aspartate transaminase and alanine aminotransferase. Several teas showed strong effects in the prevention of NAFLD, which could be developed into functional foods against NAFLD.

## Graphical abstract

The effects of 12 tea extracts on NAFLD were assessed and compared.Several teas showed strong effects in the prevention of NAFLD, which could be developed into functional foods against NAFLD.

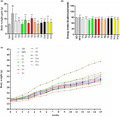

## 1 INTRODUCTION

Nonalcoholic fatty liver disease (NAFLD) severely threatens human health with a high prevalence of 25.24% across the globe, which causes huge economic and clinical burdens (Mitra et al., 2020). In addition, NAFLD could be developed into fibrosis and cirrhosis, which contributes to liver failure or hepatocellular carcinoma in the end (Tiniakos et al., 2010). The characteristic of NAFLD is known as the accumulation of excessive triglyceride (TG) in hepatocytes (hepatic TG > 5% of liver weight) (M. H. Pan et al., 2014). Thus, the prevention of NAFLD is important for the control and management of the liver disease. Additionally, overweight and obese people are more susceptible to NAFLD (Stefan et al., 2019). Obesity is closely associated with the development of NAFLD (Meng et al., 2018). Excessive intake of dietary fat and energy could increase the TG store in adipocytes, which results in obesity. The lipolysis of adipose tissue increases the level of free fatty acids (FFAs), which could promote the synthesis of TG in the liver (Pan et al., 2014). Meanwhile, overloaded FFAs are oxidized in the liver, which causes excessive production of reactive oxygen species (ROS), resulting in oxidative stress (Shin et al., 2018). Furthermore, oxidative stress can give rise to lipid peroxidation as well as hepatocyte injury, thus promoting the progress of NAFLD (Pan et al., 2014). Therefore, some natural products which possess strong anti‐obesity and/or antioxidant activity could be a good alternative for the prevention of NAFLD because of the limited efficacy and potential side effects of chemical drugs.

Tea (*Camellia sinensis*) is famous all over the world and its unique taste makes it a popular drink. Tea has multiple biological activities, such as antioxidant, anti‐cancer, anti‐obesity, and antidiabetic properties, because it is abundant in bioactive compounds such as catechins (Cao et al., 2019; Meng et al., 2019; Xu et al., 2020; Zhao et al., 2019). Catechins mainly includes epigallocatechin gallate (EGCG), gallocatechin gallate (GCG), gallocatechin (GC), epigallocatechin (EGC), epicatechin (EC), epicatechin gallate (ECG), catechin (C), and catechin gallate (CG). Due to the diverse fermentation degrees, tea can be categorized as white, green, yellow, oolong, black, and dark teas (Tang et al., 2019). A meta‐analysis showed that green tea consumption could decrease the risk of NAFLD (Yin et al., 2015). Moreover, a clinical trial demonstrated that 700 ml/day of green tea could decrease the body fat as well as improved liver function of patients with NAFLD (Sakata et al., 2013). In addition, the treatment of epigallocatechin gallate (EGCG) alleviated obesity as well as hepatic steatosis in a mouse model induced by high‐fat diet (HFD) (Bose et al., 2008). However, previous studies generally focused on one category of tea. This research was designed to assess and compare the effects of 12 kinds of teas selected from six categories on NAFLD induced by HFD. Most of these teas were firstly evaluated for their effects against NAFLD. The findings might provide useful information for the development of dietary supplements or functional foods against NAFLD, and guidance for the public to choose the most effective tea for the prevention and management of NAFLD.

## 2 MATERIALS AND METHODS

### 2.1 Chemicals

Formic acid, isopropanol, and methanol used for HPLC were offered by Macklin Chemical Factory (Shanghai, China). A total of 18 kinds of standard components used for HPLC test were purchased from Derick Biotechnology Co., Ltd. (Chengdu, China), which includes kaempferol, astragalin, quercitrin, theaflavin, quercetin, myricetin, ellagic acid, caffeine, gallic acid, chlorogenic acid, gallocatechin (GC), gallocatechin gallate (GCG), epigallocatechin (EGC), catechin (C), epigallocatechin gallate (EGCG), epicatechin (EC), catechin gallate (CG), and epicatechin gallate (ECG).

### 2.2 Extraction of teas

Table 1 showed the details of 12 kinds of teas, including the category, fermentation degree, and production place. The extraction process of tea was based on the published literature (Cao et al., 2020). Firstly, 10 g of tea was placed in 100‐mL boiling deionized water and extracted in a 98°C water bath for 10 min. The tea leave was extracted for three times and the tea extracts would be collected and filtered. Then, the tea extracts could be concentrated to about 15 ml by a vacuum rotary evaporator at 60°C. The concentrated infusions were frozen into powders by a lyophilizer in the end. The powder of tea extracts was kept at − 80°C, and it was dissolved in deionized water to obtain tea extracts with a concentration of 20 g/L (w/v) when needed.

**TABLE 1:** The detailed information of 12 kinds of teas

No	Name	Category	Fermentation Degree	Production Place
T1	Gongmei White Tea	White Tea	Mild‐fermented	Fuzhou, Fujian
T2	White Peony Tea	White Tea	Mild‐fermented	Fuzhou, Fujian
T3	Enshi Yulu Tea	Green Tea	Unfermented	Enshi, Hubei
T4	Fried Green Tea	Green Tea	Unfermented	Shaoxing, Zhejiang
T5	Yihong Tea	Black Tea	Deep‐fermented	Yichang, Hubei
T6	Lapsang Souchong Tea	Black Tea	Deep‐fermented	Xiamen, Fujian
T7	Wuyi Narcissus Tea	Oolong Tea	Semi‐fermented	Wuyishan, Fujian
T8	Fenghuang Narcissus Tea	Oolong Tea	Semi‐fermented	Shantou, Guangdong
T9	Qing Brick Tea	Dark Tea	Post‐fermented	Yichang, Hubei
T10	Pu‐erh Tea	Dark Tea	Post‐fermented	Pu'er, Yunnan
T11	Yuan'an Luyuan Tea	Yellow Tea	Light‐fermented	Yichang, Hubei
T12	Mengding Huangya Tea	Yellow Tea	Light‐fermented	Mengdingshan, Sichuan

### 2.3 Animal study

C57BL/6J male mice (18–20 g) were obtained from the Experimental Animal Center of Guangdong province, Guangzhou, China. The experimental protocols of mice used within this study complied with the ethical guidelines suggested by the Animal Care Committee at the School of Public Health, Sun Yat‐Sen University (No. 2019–002; 28 February 2019). The mice were housed in a room of specific pathogen‐free condition and the room kept at temperature of 22 ± 0.5°C and humidity of 40%–60% with a 12‐hr light/dark cycle. After one‐week acclimatization, 8‐week‐old mice were randomly divided into 14 groups (*n* = 10), which includes a normal diet (ND) control, a HFD model group, and 12 tea‐treatment groups. The mice in the control group received a ND (3.6 kcal/g, 12% calories from fat), which was obtained from Jiangsu Xietong Pharmaceutical Bioengineering Co., Ltd. (Nanjing, China). The other groups were fed with HFD (5.0 kcal/g, 60% calories from fat, TP 23,400), which was obtained from TROPHIC Animal Feed High‐tech Co., Ltd. (Nantong, China). High doses of EGCG might cause liver injury. According to some published literature, the intervention groups in our study were administrated with 200 mg/(kg∙d) tea extracts for 15 weeks by intragastric gavage (Bae et al., 2018; Liu et al., 2019). Meanwhile, the control and model groups were given deionized water (10 ml/kg) intragastrically. All mice had free access to diet as well as water. The consumption of food as well as body weight was recorded daily and weekly, respectively. When the experiments come to end, all mice were weighed and anesthetized after fasting for 12 hr. Then removing the eyeballs of mice, the blood samples were obtained and stored at 25°C for 1 hr. Then, the blood samples were centrifuged at the condition of 3,000 × g as well as 4°C for 15 min to obtain the serum, which was kept at 4°C before further biochemical assays. Moreover, the liver, perirenal adipose tissue as well as epididymal adipose tissue were harvested. A part of the liver as well as a piece of epididymal adipose tissue were cut for histopathological analysis, and the rest of liver was kept at − 80°C for further use.

### 2.4 Biochemical analysis of serum

The contents of AST, ALT, TC, TG as well as LDL‐C in serum were measured by kits obtained from Roche diagnostics (Shanghai, China). Briefly, the contents of TG, TC as well as LDL‐C were determined via enzymatic tests. Additionally, the contents of AST as well as ALT were detected through velocity tests.

### 2.5 Determination of hepatic TG and total protein contents

The 25 mg of hepatic tissue was mixed with 500 μL of lysis buffer, and then the mixture was homogenized. The supernatant of liver homogenate was heated at 70°C for 10 min, and the product was centrifuged to obtain the supernatant (2,000 × g, 25°C, and 5 min). The detection kits of hepatic TG and total protein were obtained from Apply‐gen Technologies Inc (Beijing, China).

### 2.6 Determination of GSH, SOD as well as MDA levels in liver

The liver tissue was mixed and homogenized with 0.9% NaCl to prepare 10% (w/v) hepatic homogenate. The liver homogenate was centrifuged at the condition of 2,500 × gas wells at 4°C for 10 min, and the supernatant was taken for the detection of glutathione and superoxide dismutase. We purchased the kits of GSH and SOD from Nanjing Jiancheng Bioengineering Institute (Nanjing, China). The detection kits of MDA were obtained from Apply‐gen Technologies Inc (Beijing, China).

### 2.7 Histopathological analysis

A part of the liver as well as a piece of epididymal adipose tissue was fixed by 4% paraformaldehyde for a few days before embedding in paraffin. Subsequently, the embedded samples could be sliced to obtain 5‐µm‐thick sections, which were deparaffinized, rehydrated, and finally stained with hematoxylin and eosin. At last, a light microscope was used for the image capturing of the liver and epididymal adipose tissue.

### 2.8 Statistical analysis

The data and values were shown as mean ± standard deviation (*SD*). Comparisons of statistical significance between groups were conducted via one‐way analysis of variance plus a posthoc least significant difference test with SPSS version 25.0 (IBM SPSS, USA). The statistical significant criteria set to be *p*‐value less than 0.05.

## 3 RESULTS AND DISCUSSION

### 3.1 Effects of tea on body weight

**FIGURE 1:**
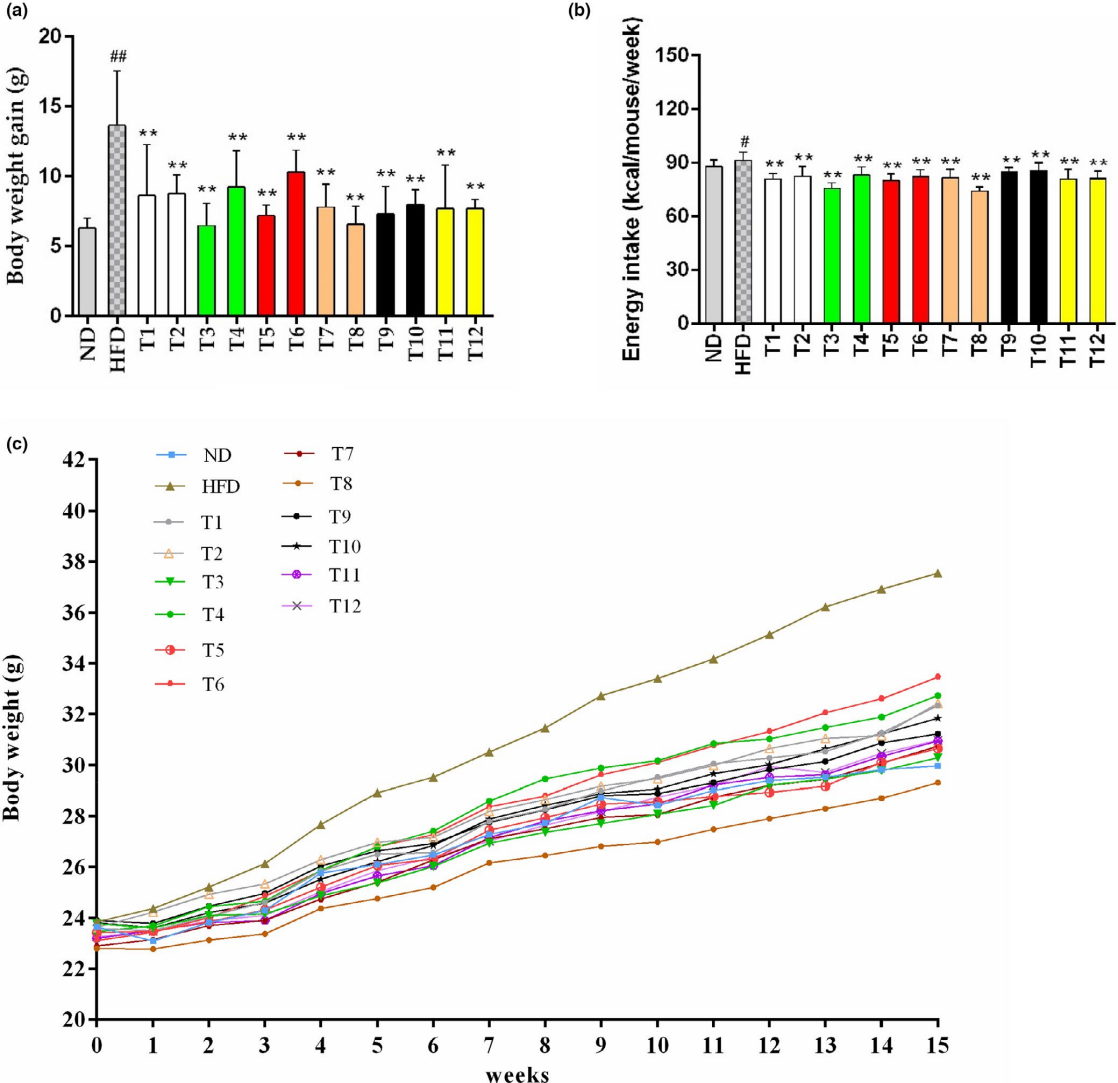
The effects of 12 types of teas on energy intake as well as body weight for 15 weeks. (a) Body weight gain; (b) Energy intake; and (c) Change in body weight for 15 weeks. Data in figures a to b are shown as means ± standard deviation (*SD*). * *p* < .05, ** *p* < .01, tea treatment versus HFD; # *p* < .05, ## *p* < .01, HFD versus ND. ND, normal diet; HFD, high‐fat diet; T1, Gongmei White Tea; T2, White Peony Tea; T3, Enshi Yulu Tea; T4, Fried Green Tea; T5, Yihong Tea; T6, Lapsang Souchong Tea; T7, Wuyi Narcissus Tea; T8, Fenghuang Narcissus Tea; T9, Qing Brick Tea; T10, Pu‐erh Tea; T11, Yuan'an Luyuan Tea; T12, Mengding Huangya Tea

The effects of 12 teas on energy intake as well as body weight in mice receiving HFD are shown in Figure 1a–c. In comparison with the mice receiving ND, the final body weight of the HFD model group increased by 25.29%, indicating that the mice had developed obesity. In addition, different teas at a dose of 200 mg/kg b.w. revealed different effects on inhibiting body weight gain (Figure 1a). Notably, 52.48% and 51.75% reductions of body weight gain were observed in Enshi Yulu Tea (T3) group and Fenghuang Narcissus Tea (T8) group, respectively, when comparing with the HFD group. Moreover, the results demonstrated that the mice in HFD group consumed more energy than that in ND group (Figure 1b). The energy consumption of mice in all tea‐treated groups was evidently decreased when compared with the model group, which was coincident with the reduction of body weight. The results were in line with previous studies, which have been reported that the consumption of tea could reduce food and energy intake, like black tea and green tea (Du et al., 2005; Huang et al., 2014; Pan et al., 2018). Furthermore, previous studies pointed out that EGCG in teas contributed to the decrease in food consumption as well as body weight via the suppression of appetite (Kao et al., 2000).

### 3.2 Effects of tea on fat mass

**FIGURE 2:**
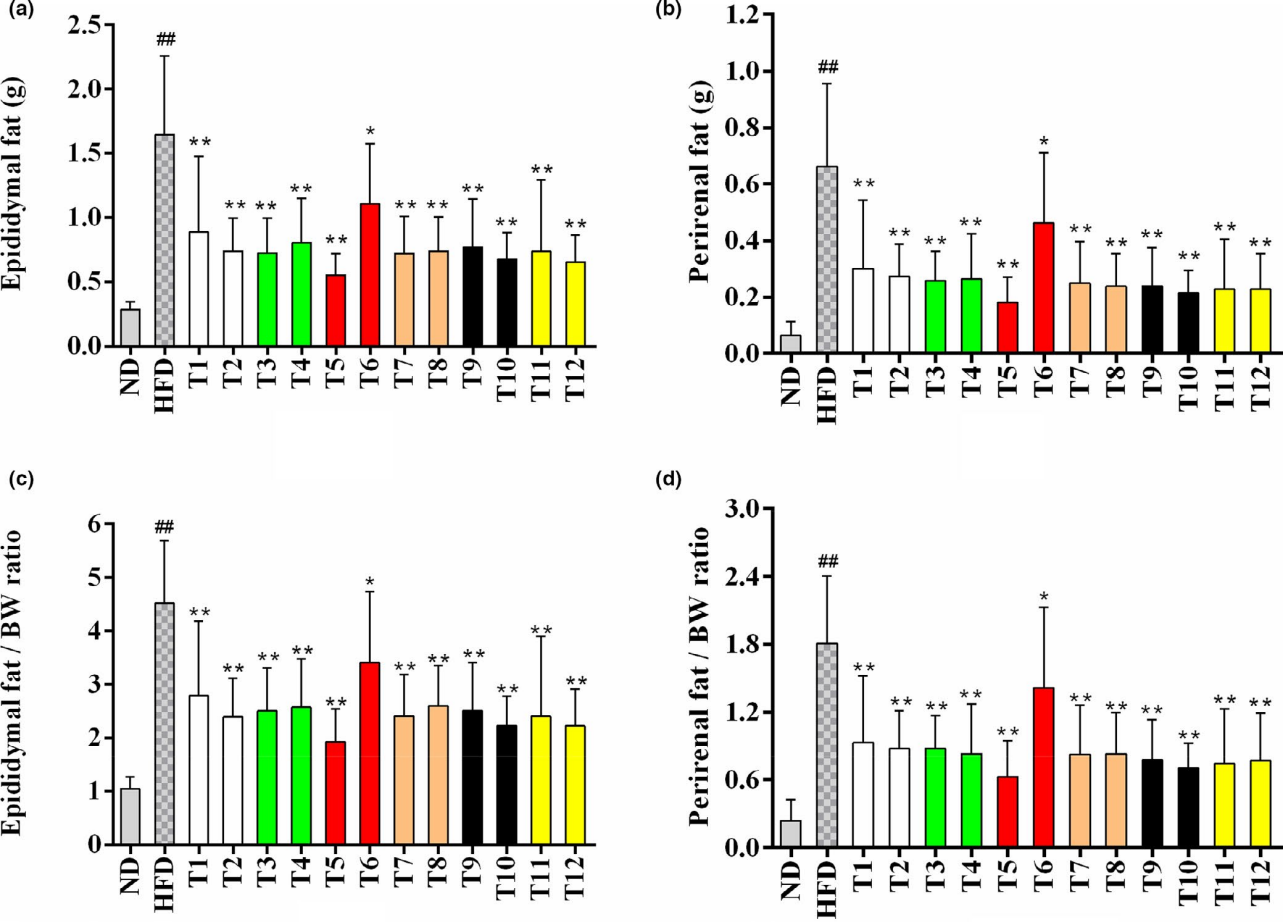
Effects of teas on fat mass. (a) Epididymal fat mass; (b) Perirenal fat mass; (c) The ratio of epididymal fat mass and body weight; (d) The ratio of perirenal fat mass and body weight. Data in figures a to b are shown as means ± *SD*. * *p* < .05, ** *p* < .01, tea treatment versus HFD; # *p* < .05, ## *p* < .01, HFD versus ND. ND, normal diet; HFD, high‐fat diet; T1, Gongmei White Tea; T2, White Peony Tea; T3, Enshi Yulu Tea; T4, Fried Green Tea; T5, Yihong Tea; T6, Lapsang Souchong Tea; T7, Wuyi Narcissus Tea; T8, Fenghuang Narcissus Tea; T9, Qing Brick Tea; T10, Pu‐erh Tea; T11, Yuan'an Luyuan Tea; T12, Mengding Huangya Tea

**FIGURE 3:**
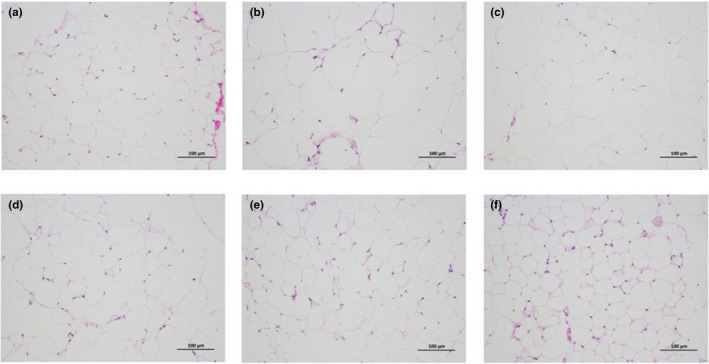
Effects of teas on epididymal adipocyte size. (a) Control group; (b) High‐fat diet group; (c) Enshi Yulu Tea group; (d) Yihong Tea group; (e) Fenghuang Narcissus Tea group; (f) Yuan'an Luyuan Tea. The histopathological images of epididymal adipose tissue (200 × magnification); scale bar = 100 μm

The results of epididymal and perirenal fats are shown in Figures 2 and 3. A 4.69‐fold and 10‐fold elevations were observed in the weight of epididymal as well as perirenal fats in the model group when compared with the ND group (Figure 2a and b). Seen from Figure 3a and b, the adipocytes of the HFD model group were more irregular and larger than that of the ND control group. Tea extracts significantly inhibited the increase of epididymal and perirenal fat mass, particularly Yihong Tea (T5) which had the lowest weight of epididymal and perirenal fat among the treatment groups. Most other teas had similarly strong efficacy in preventing adipose accumulation except Lapsang Souchong Tea (T6) which had a mild effect on decreasing fat mass (Figure 2a–d). The histopathological results showed that epididymal adipocytes in mice treated with tea extracts were smaller and arranged more regularly in comparison with the model group (Figure 3c–f). Epididymal and perirenal fats could store excessive TG, which are important visceral adipose tissues. The content of FFA in the liver was increased after the lipolysis of the visceral adipose tissue, leading to the development of NAFLD. Thus, the inhibition of fat accumulation was important for the prevention of NAFLD. The greatest decrease in epididymal and perirenal fat mass was found in the Yihong Tea group, and it might have potential effects on preventing NAFLD development.

### 3.3 Effects of tea on liver parameters

**FIGURE 4:**
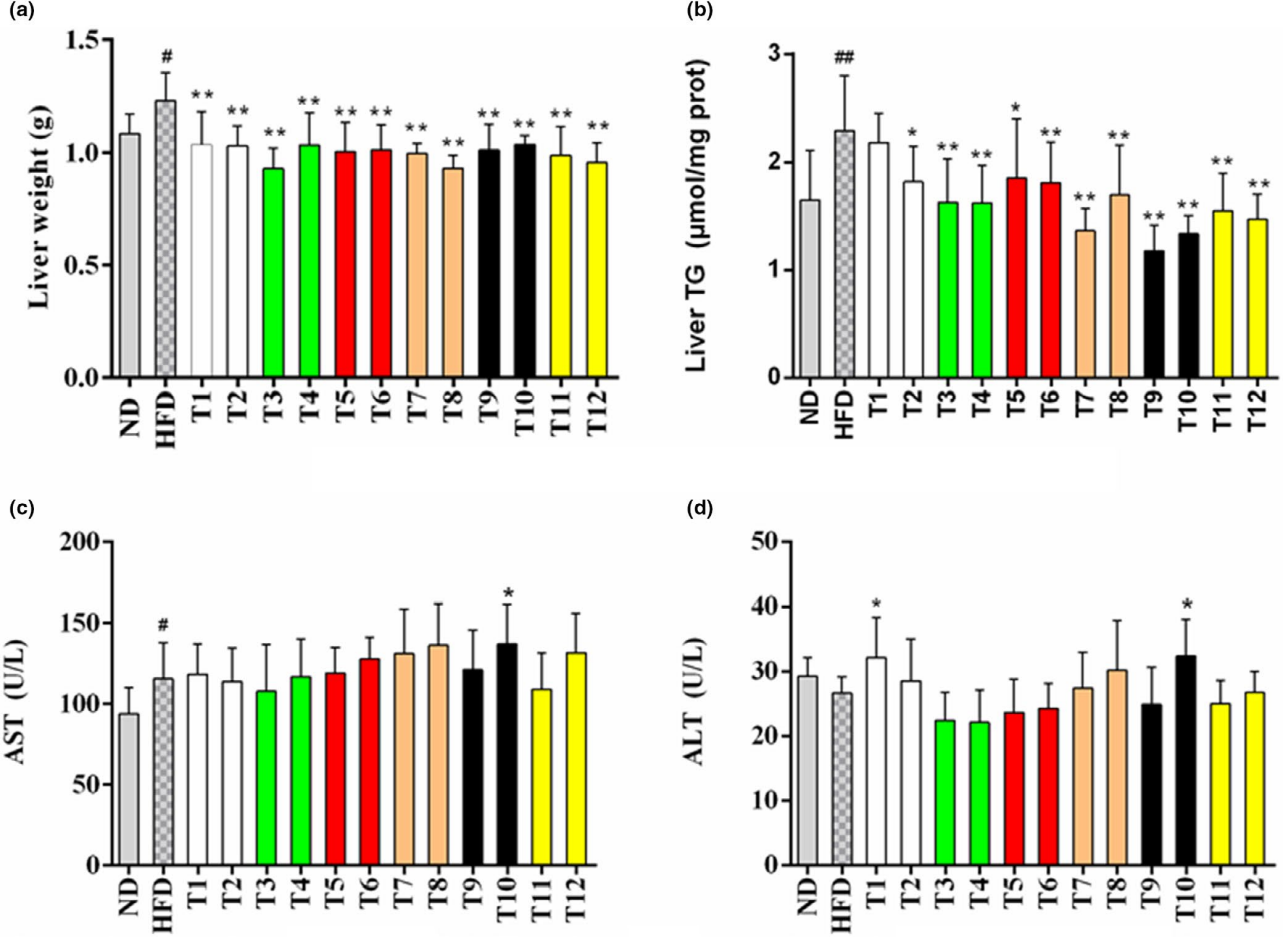
Effects of teas on liver parameters. (a) Liver weight; (b) Liver TG content, (c) AST in serum; (d) ALT in serum. The values are displayed as means ± *SD*. * *p* < .05, ** *p* < .01, tea treatment versus HFD; # *p* <.05, ## *p* < .01, HFD versus ND. TG, triglyceride; AST, aspartate transaminase; ALT, alanine aminotransferase; ND, normal diet; HFD, high‐fat diet; T1, Gongmei White Tea; T2, White Peony Tea; T3, Enshi Yulu Tea; T4, Fried Green Tea; T5, Yihong Tea; T6, Lapsang Souchong Tea; T7, Wuyi Narcissus Tea; T8, Fenghuang Narcissus Tea; T9, Qing Brick Tea; T10, Pu‐erh Tea; T11, Yuan'an Luyuan Tea; T12, Mengding Huangya Tea

Seen from Figure 4, HFD could significantly increase liver weight as well as the level of hepatic TG compared with the ND. Additionally, it was found that 12 teas could alleviate the elevation of liver weight, while the effects varied in different kinds of teas (Figure 4a). The effects were more obvious in the Enshi Yulu Tea group (T3) and Fenghuang Narcissus Tea group (T8). The result of hepatic TG content was largely consistent with that of liver weight. Several teas remarkably decreased the level of TG, except Gongmei White Tea (T1) (Figure 4b). The Qing Brick Tea (T9) was the most effective tea in preventing the accumulation of TG in the liver, which is followed by Wuyi Narcissus Tea (T7).

The results of liver function enzymes are shown in Figure 4c and d. When comparing with the ND group, the level of aspartate transaminase (AST) was significantly increased while no evident elevation of alanine aminotransferase (ALT) level was observed in the model group. The levels of AST and ALT in tea‐supplementation groups had no significant decrease when compared with those in the HFD group. However, Pu‐erh Tea (T10) increased the level of AST and ALT in comparison with the ND control group. Moreover, Gongmei White Tea (T1) also elevated the level of ALT in comparison with the HFD group. ALT as well as AST are two important liver function enzymes reflecting hepatocellular injury (Kunde et al., 2005). The results indicated that most teas did not exhibit hepatotoxicity, while Pu‐erh Tea and Gongmei White Tea might cause damage in the hepatocytes of mice at the dosage of 200 mg/kg b.w.

### 3.4 Histopathological evaluation of liver

**FIGURE 5:**
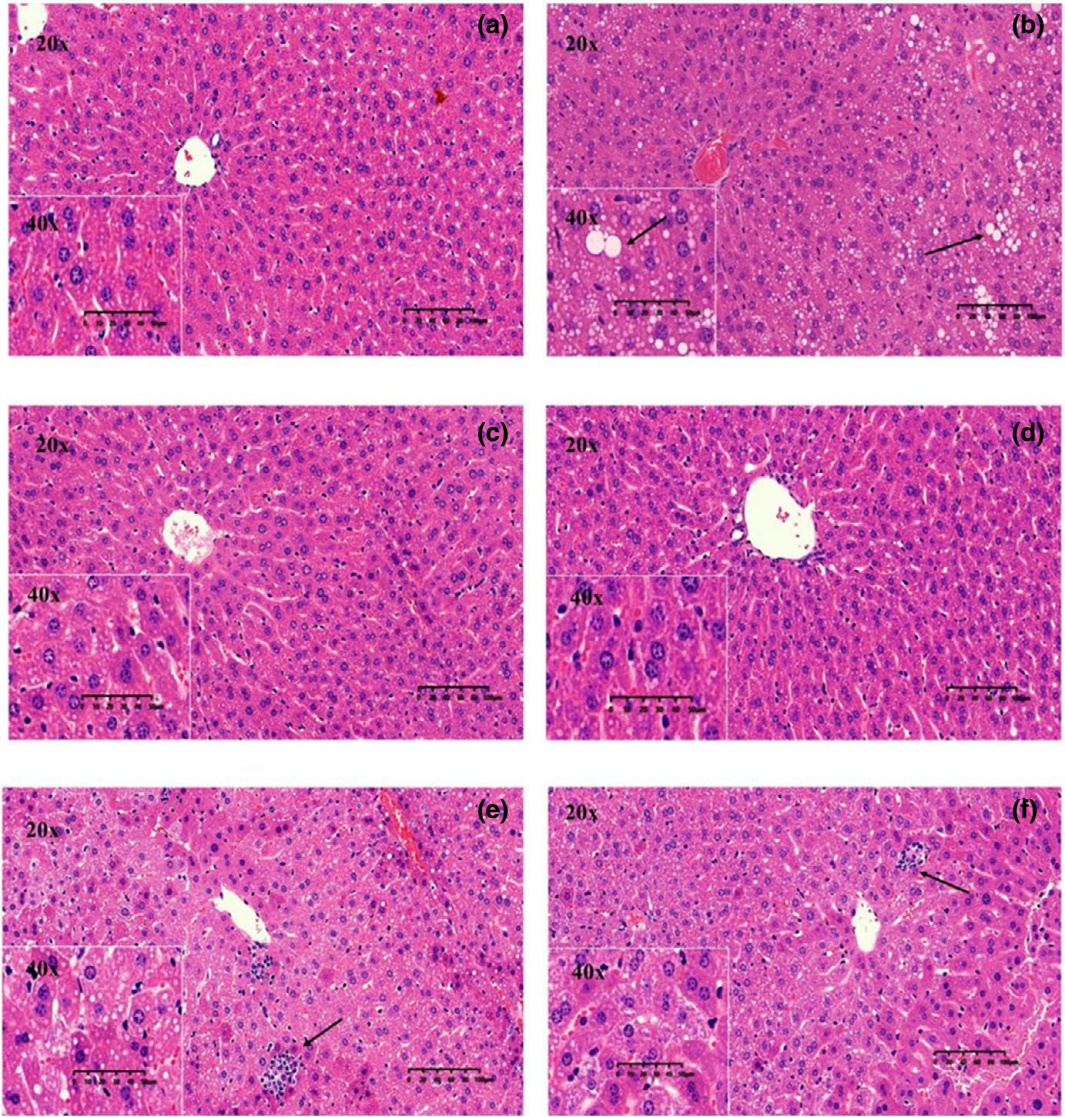
The histopathological images of hepatic tissue (200 × and 400 × magnification). (a) Normal diet group; (b) High fat diet group; (c) Enshi Yulu Tea group; (d) Qing Brick Tea group; (e) White Peony Tea group; (f) Pu‐erh Tea group

The preventive effect of several tea treatments against hepatic steatosis was further verified by histopathological assay (Figure 5). No obvious damage was found in the liver of ND group (Figure 5a). However, in HFD fed mice, the hepatocytes did not arrange orderly and there was extensive hepatocyte steatosis with many lipid droplets, indicating that simple liver steatosis occurred (Figure 5b). As displayed in Figure 5c and d, tea supplementation significantly attenuated the accumulation of lipid droplets as well as alleviated hepatic steatosis led by the HFD. However, Figure 5e as well as Figure 5f revealed that inflammatory cell aggregation was seen in White Peony Tea and Pu‐erh Tea groups.

### 3.5 Effects of tea on serum lipid profiles

**FIGURE 6:**
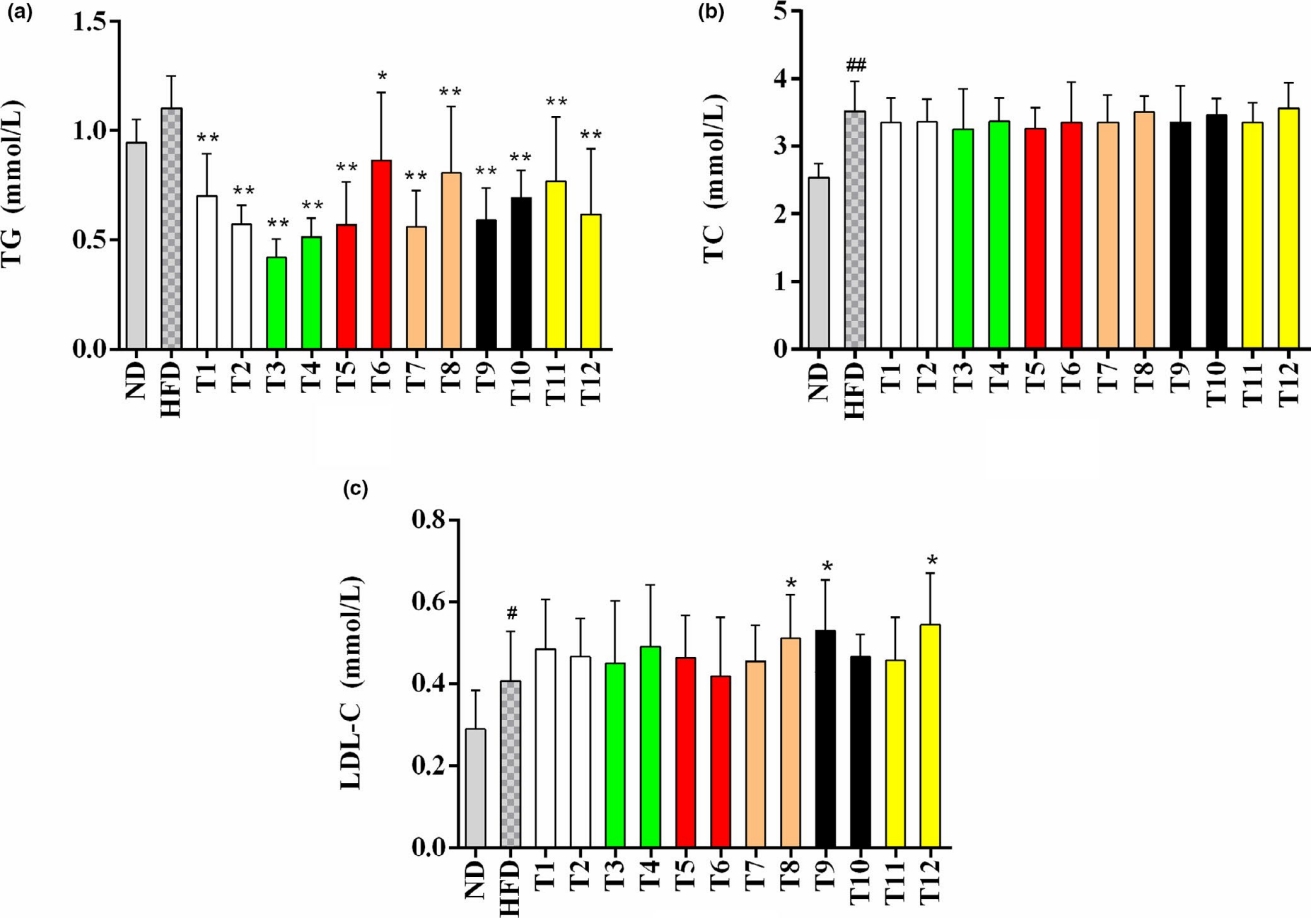
Effects of 12 kinds of teas on serum lipid profiles. (a) TG; (b) TC; (c) LDL‐C. The values are displayed as means ± *SD*. * *p* < .05, ** *p* < .01, tea treatment versus HFD; # *p* < .05, ## *p* < .01, HFD versus ND. TG, triglyceride; TC, total cholesterol; LDL‐C, low‐density lipoprotein cholesterol; ND, normal diet; HFD, high‐fat diet; T1, Gongmei White Tea; T2, White Peony Tea; T3, Enshi Yulu Tea; T4, Fried Green Tea; T5, Yihong Tea; T6, Lapsang Souchong Tea; T7, Wuyi Narcissus Tea; T8, Fenghuang Narcissus Tea; T9, Qing Brick Tea; T10, Pu‐erh Tea; T11, Yuan'an Luyuan Tea; T12, Mengding Huangya Tea

The results of serum TG, total cholesterol (TC), and low‐density lipoprotein cholesterol (LDL‐C) levels are displayed in Figure 6. In comparison with the ND control group, the level of serum TG was not evidently elevated in mice receiving HFD. However, all tea extracts obviously attenuated the increase of TG level in serum, especially the green teas (T3 and T4) with a reduction of 61.82% and 52.73%, respectively (Figure 6a) when comparing with the HFD group. In addition, the results demonstrated that HFD‐feeding markedly increased the levels of TC and LDL‐C (Figure 6b and c). Although the treatment of tea extracts did not show significant effects in lowering the level of serum TC, most teas had a decreasing tendency in serum TC level compared with the model group. However, the level of LDL‐C was elevated in the groups administrated with Fenghuang Narcissus (T8), Qing Brick (T9), and Mengding Huangya (T12) teas when comparing with the HFD model group.

Previous researches reported that obesity and NAFLD could affect lipid metabolism as well as contribute to hypercholesterolemia and hypertriglyceridemia (Gaggini et al., 2013; Shin & Jung, 2017; Vekic et al., 2019). Several studies suggested that the supplementation of tea could lead to the decrease in the level of serum TG (Cao et al., 2011; Chen et al., 2018). However, the effects of tea on lowering serum TC and LDL‐C were controversial based on the results from different studies, which might be due to the difference in duration and dose of tea treatment. For example, a clinical trial revealed that green tea treatment did not significantly decrease the level of LDL in patients with NAFLD (Rostampour et al., 2019). In an obese rat model, the levels of TC as well as LDL‐C could be evidently reduced by 12‐week oral gavage of green tea extract (500 mg/kg b.w.) (Rocha et al., 2016). Overall, the findings of our study indicated that some tea extracts could improve the elevated TG level in serum and might prevent the onset of NAFLD.

### 3.6 Effects of tea on liver redox state

As displayed in Table 2, the HFD group showed an obvious decrease in the content of glutathione (GSH) when compared with the ND control group. The content of GSH was significantly decreased by Fried Green Tea and Yihong Tea treatment, while Fenghuang Narcissus Tea increased the content of GSH when comparing with the HFD model group. Moreover, the activity of superoxide dismutase (SOD) was higher in HFD‐feeding mice than that in the mice of ND control group. There was an evident decrease in SOD activity in Enshi Yulu Tea, Fried Green Tea, Yihong Tea, Lapsang Souchong Tea, and Qing Brick Tea groups when comparing with the HFD group. Additionally, no marked difference was found in the level of malondialdehyde (MDA) among the control, model and treatment groups. Oxidative stress is thought to be the main cause of lipid peroxidation, which is crucial for the progression of NAFLD (J. H. Shin & Jung, 2017; Valenti et al., 2013). MDA is the hallmark of lipid peroxidation and the content of MDA could reflect the redox state to some extent. SOD and GSH are considered as an important enzymatic and non‐enzymatic antioxidant, respectively (Leung & Nieto, 2013). The activity of SOD could be elevated and the level of MDA could be reduced by 0.5%–1% green tea extract in a leptin‐deficient (ob/ob) mouse model (Park et al., 2011). However, our findings were not in accordance with some studies. The content of MDA was not markedly elevated in the HFD group, we were unclear whether it resulted from the activation of SOD. We speculated that two factors might account for the inconsistency. On the one hand, liver antioxidant enzymes were activated in the model group when the oxidative stress existed (Perlemuter et al., 2005). The low activity of SOD in the treatment groups meant that tea extracts might defend against ROS through the antioxidant property of the polyphenols like free radical‐scavenging ability and ferric‐reducing antioxidant power (Zhao et al., 2019). On the other hand, the TG content in the treatment group was low, indicating that the oxidation of FFA in tea‐treatment group was less than that in the HFD group and less ROS was produced. Thus, the activity of SOD was decreased in the groups treated with tea extracts in comparison with the HFD group. In conclusion, Fenghuang Narcissus Tea, Enshi Yulu Tea, and Qing Brick Tea might have in vivo antioxidant property, which were critical for the prevention of NAFLD.

**TABLE 2:** Effects of 12 teas on liver redox state

Group	GSH (μmol/g prot)	SOD (U/mg prot)	MDA (nmol/mg prot)
Normal diet (ND)	5.26 ± 2.10	110.72 ± 15.34	0.38 ± 0.08
High fat diet (HFD)	3.94 ± 1.14**#**	135.52 ± 15.66**##**	0.47 ± 0.13
Gongmei White Tea (T1)	3.46 ± 0.79	124.29 ± 17.52	0.46 ± 0.05
White Peony Tea (T2)	3.76 ± 0.92	127.39 ± 7.68	0.43 ± 0.10
Enshi Yulu Tea (T3)	3.82 ± 0.40	115.68 ± 9.34**	0.43 ± 0.09
Fried Green Tea (T4)	2.58 ± 0.65*	105.60 ± 18.29**	0.46 ± 0.11
Yihong Tea (T5)	2.16 ± 0.37**	105.94 ± 14.19**	0.42 ± 0.09
Lapsang Souchong Tea (T6)	4.63 ± 1.90	118.91 ± 15.39*	0.38 ± 0.12
Wuyi Narcissus Tea (T7)	4.91 ± 1.62	123.69 ± 12.56	0.39 ± 0.10
Fenghuang Narcissus Tea (T8)	5.30 ± 0.98*	132.37 ± 19.78	0.45 ± 0.12
Qing Brick Tea (T9)	3.80 ± 1.07	118.51 ± 11.18*	0.38 ± 0.20
Pu‐erh Tea (T10)	4.23 ± 0.76	126.47 ± 14.88	0.44 ± 0.15
Yuan'an Luyuan Tea (T11)	4.53 ± 1.36	136.17 ± 15.88	0.40 ± 0.09
Mengding Huangya Tea (T12)	4.37 ± 1.38	128.57 ± 20.39	0.42 ± 0.16

Note

The values are displayed as means ± *SD*.

Abbreviations: GSH, glutathione; MDA, malondialdehyde; SOD, superoxide dismutase.

*p < .05, **p < .01, tea treatment versus HFD; #*p* < .05, ##*p* < .01, HFD versus ND.

### 3.7 Bioactive components of teas

**FIGURE 7:**
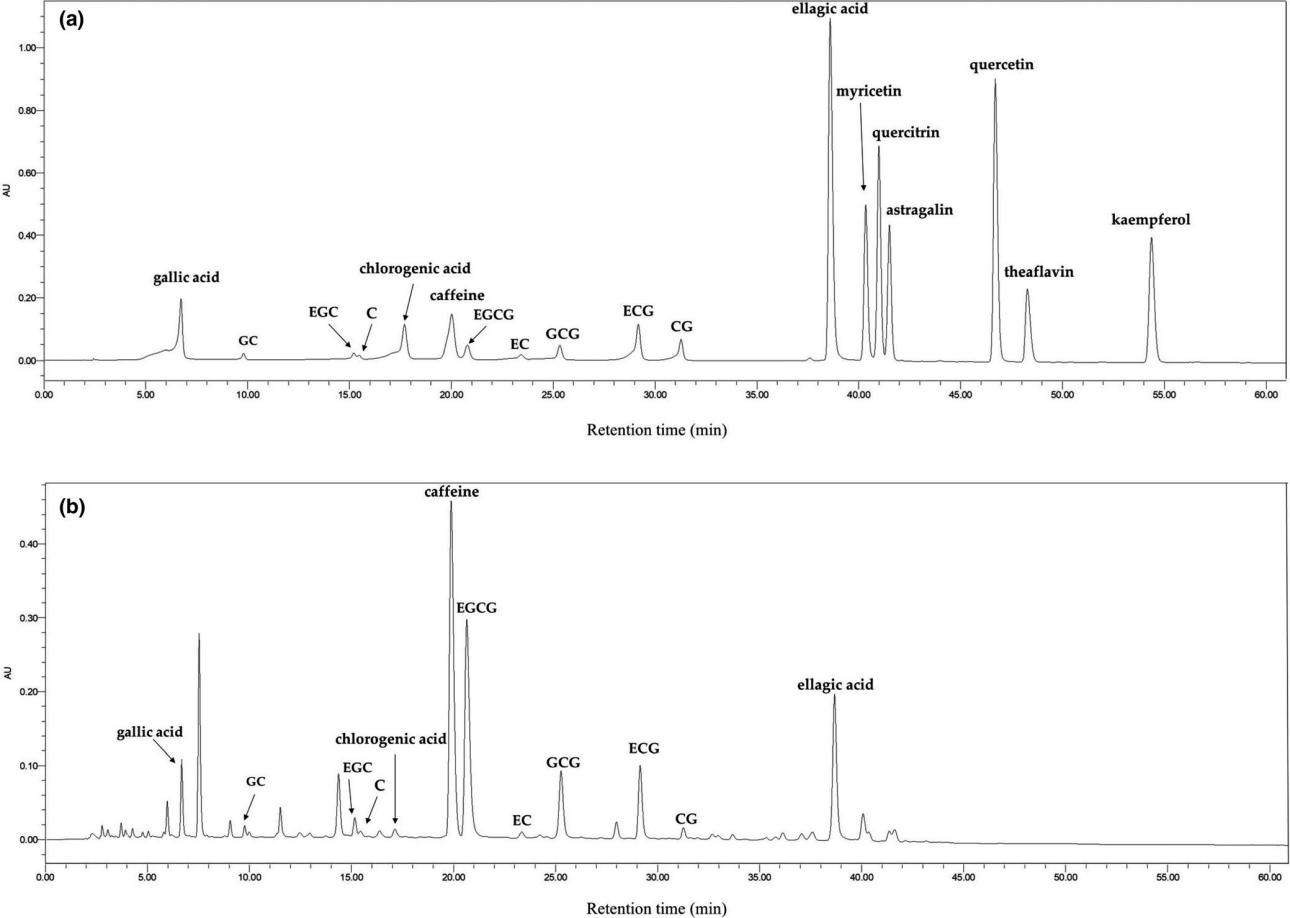
The HPLC chromatograms of the standard components (a) as well as Enshi Yulu Tea (b) in 254 nm. GC, gallocatechin;,EGC, epigallocatechin; C, catechin; EGCG, epigallocatechin gallate; EC, epicatechin; GCG, gallocatechin gallate; ECG, epicatechin gallate; CG, catechin gallate

HPLC method was used for the measurement of main phytoconstituents in 12 kinds of tea extracts. Figure 7a and b displayed the chromatograms of standard components and Enshi Yulu Tea at 254 nm. The results of main constituents in 12 teas are displayed in Table 3. There were 13 phytochemicals determined and quantified in tea extracts, which includes eight types of catechins and five other active constituents (astragalin, caffeine, chlorogenic acid, ellagic acid, and gallic acid). In addition, caffeine and gallic acid were identified in all tested teas. Most teas possessed ellagic acid, astragalin, chlorogenic acid, EGCG, GCG, GC, EGC, EC, and ECG, but the contents were greatly different. However, C and CG only existed in two kinds of teas.

**TABLE 3:** The contents (mg/g DW) of major bioactive compounds in12 kinds of teas

Name	Gallocatechin	Epigallocatechin	Catechin	Epigallocatechin Gallate	Epicatechin	Gallocatechin Gallate	Epicatechin Gallate
Gongmei White Tea (T1)	–	29.14 ± 0.79	–	186.24 ± 3.76	9.42 ± 0.23	–	15.97 ± 0.92
White Peony Tea (T2)	–	–	–	206.97 ± 5.86	6.84 ± 0.16	–	19.11 ± 0.28
Enshi Yulu Tea (T3)	21.71 ± 0.43	26.75 ± 0.34	24.43 ± 0.34	258.35 ± 4.05	12.24 ± 0.33	63.37 ± 2.22	23.49 ± 0.28
Fried Green Tea (T4)	137.53 ± 6.19	172.3 ± 5.05	–	566.62 ± 21.64	45.51 ± 1.42	137.48 ± 6.06	39.41 ± 1.34
Yihong Tea (T5)	–	–	–	8.99 ± 0.73	–	–	–
Lapsang Souchong Tea (T6)	–	–	–	–	–	–	–
Wuyi Narcissus Tea (T7)	54.03 ± 1.22	39.58 ± 0.80	23.05 ± 0.95	153.22 ± 3.68	12.28 ± 0.32	39.52 ± 1.38	11.89 ± 0.76
Fenghuang Narcissus Tea (T8)	20.98 ± 0.55	32.65 ± 0.35	–	394.18 ± 3.04	10.42 ± 0.18	64.74 ± 3.26	31.90 ± 0.53
Qing Brick Tea (T9)	8.72 ± 0.61	–	–	57.18 ± 1.25	7.90 ± 0.11	–	–
Pu‐erh Tea (T10)	–	–	–	–	3.98 ± 0.08	–	–
Yuan'an Luyuan Tea (T11)	12.58 ± 0.56	17.74 ± 0.45	–	331.55 ± 8.98	13.23 ± 0.32	66.13 ± 2.23	32.88 ± 1.19
Mengding Huangya Tea (T12)	8.68 ± 0.29	13.56 ± 0.43	–	122.05 ± 1.21	9.38 ± 0.56	–	13.44 ± 0.39

Name	Catechin Gallate	Gallic Acid	Chlorogenic Acid	Caffeine	Ellagic Acid	**Astragalin**
Gongmei White Tea (T1)	–	11.92 ± 0.26	–	99.69 ± 1.01	2.29 ± 0.09	–
White Peony Tea (T2)	–	15.57 ± 0.60	2.39 ± 0.02	96.84 ± 2.00	1.91 ± 0.05	4.15 ± 0.20
Enshi Yulu Tea (T3)	6.93 ± 0.26	10.06 ± 0.54	1.28 ± 0.02	90.20 ± 0.99	8.12 ± 0.24	–
Fried Green Tea (T4)	–	11.84 ± 0.45	7.79 ± 0.03	233.19 ± 2.58	–	20.34 ± 0.79
Yihong Tea (T5)	–	25.50 ± 0.93	–	84.51 ± 0.96	4.69 ± 0.15	2.55 ± 0.14
Lapsang Souchong Tea (T6)	–	25.69 ± 0.85	–	80.43 ± 1.16	2.67 ± 0.13	2.88 ± 0.15
Wuyi Narcissus Tea (T7)	–	16.88 ± 0.49	–	84.90 ± 1.21	1.75 ± 0.09	1.90 ± 0.09
Fenghuang Narcissus Tea (T8)	–	20.12 ± 0.58	1.74 ± 0.02	101.30 ± 0.84	3.95 ± 0.15	7.00 ± 0.23
Qing Brick Tea (T9)	–	31.75 ± 0.56	–	92.28 ± 0.87	2.13 ± 0.18	–
Pu‐erh Tea (T10)	–	11.98 ± 0.38	1.11 ± 0.01	94.02 ± 0.64	3.52 ± 0.22	–
Yuan'an Luyuan Tea (T11)	–	19.18 ± 0.95	2.88 ± 0.03	105.46 ± 1.76	13.31 ± 0.35	–
Mengding Huangya Tea (T12)	3.12 ± 0.14	12.93 ± 0.45	–	62.33 ± 0.46	9.64 ± 0.19	–

Abbreviations: –, means not detected;DW, dry weight of tea extracts.

The results showed that catechins are abundant in teas, and EGCG was the richest catechin with a range from 8.99 ± 0.73 to 566.62 ± 21.64 mg/g DW (dry weight of tea extracts). The content of EGCG reached up to 566.62 ± 21.64 mg/g DW in Fried Green Tea, and the content of EGCG in Fenghuang Narcissus Tea was second to it with the value of 394.18 ± 3.04 mg/g DW.

Additionally, gallocatechin was found in seven kinds of teas with values ranging from 8.68 ± 0.29 to 137.53 ± 6.19 mg/g DW. Moreover, most teas found epicatechin which ranged from 3.98 ± 0.08 to 45.51 ± 1.42 mg/g DW. There were eight kinds of teas possessing epigallocatechin with the contents ranging from 13.56 ± 0.43 to 172.3 ± 5.05 mg/g DW. Furthermore, catechin was found in Enshi Yulu Tea and Wuyi Narcissus Tea with the contents of 24.43 ± 0.34 as well as 23.05 ± 0.95 mg/g DW, respectively. Catechin gallate was only detected in two kinds of teas and the contents were low.

Apart from catechins, caffeine was rich in tea extracts with the contents from 62.33 ± 0.46 to 233.19 ± 2.58 mg/g DW. Fried Green Tea (233.19 ± 2.58 mg/g DW) had the highest content of caffeine, followed by Yuan'an Luyuan Tea (105.46 ± 1.76 mg/g DW). Besides, the contents of gallic acid covered from 10.06 ± 0.54 to 31.75 ± 0.56 mg/g DW. Qing Brick Tea possessed the highest content of gallic acid. Moreover, ellagic acid was detected in all tea extracts except Fried Green Tea with the values from 1.75 ± 0.09 to 13.31 ± 0.35 mg/g DW. Furthermore, the tested tea extracts contained a small amount of chlorogenic acid with the values from to 1.11 ± 0.01 to 7.79 ± 0.03 mg/g DW. Astragalin was found in six kinds of tea extracts, among which Fried Green Tea had the highest content (20.34 ± 0.79 mg/g DW). In general, Fried Green Tea had high contents of phytochemicals and Enshi Yulu Tea contained the most abundant kinds of active compounds. The fermentation degrees of teas might be accounted for the results, since Fried Green Tea and Enshi Yulu Tea were non‐fermented green teas. It has been reported that manufacturing process could change the chemical composition of teas (Jiang et al., 2019).

**FIGURE 8:**
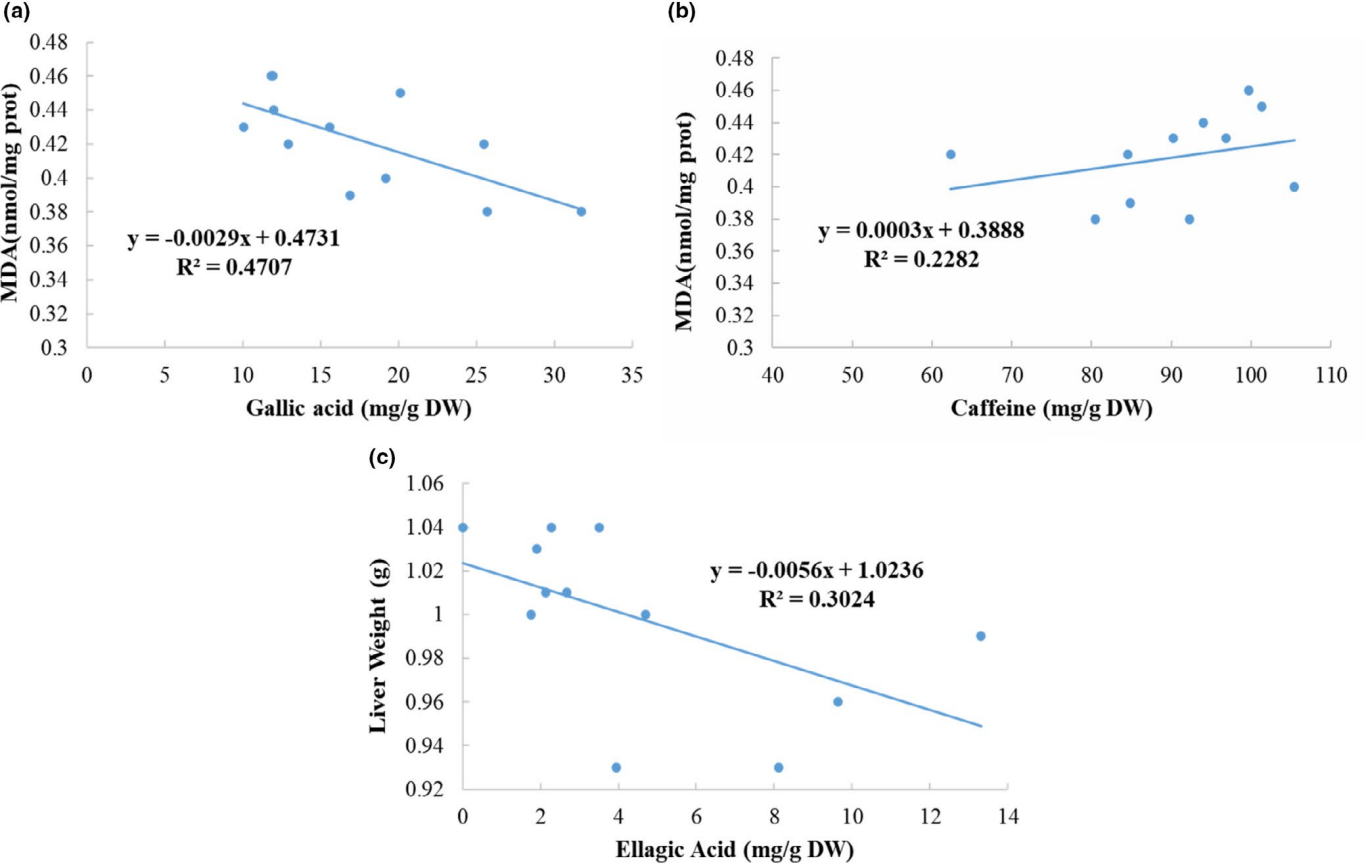
Correlations between (a) gallic acid and MDA; (b) caffeine and MDA; (c) ellagic acid and liver weight

We conducted a correlation analysis of 13 biochemical indicators and 13 phytochemicals in teas to investigate the associations of the bioactive compounds and the biological activities. The results demonstrated the content of gallic acid had a negative association with the level of MDA with the R^2^ value of 0.4707 (Figure 8a). However, the content of caffeine had a positive relation with the level of MDA with the R^2^ value of 0.2282 (Figure 8b). Moreover, there was a negative relationship between ellagic acid and liver weight, and the R^2^ value was 0.3024 (Figure 8c). Furthermore, no evident associations were observed between other constituents and biochemical indicators. Generally, the results indicated that gallic acid might improve oxidative stress through decreasing the content of MDA. Besides, high content of caffeine might induce oxidative stress by increasing the level of MDA. Additionally, ellagic acid might reduce liver weight. The results were consistent with some studies. For example, a study found that the treatment of caffeine could increase the level of MDA in the zebrafish brain (de Carvalho et al., 2019). Several studies showed that ellagic acid and gallic acid could ameliorate liver steatosis in mice (Sousa et al., 2020; Zhang et al., 2019).

## 4 CONCLUSIONS

In our research, we assessed and compared the effects of 12 teas on NAFLD in mice led by a HFD. It was found that several tea extracts could prevent NAFLD through significantly decreasing energy consumption, body weight gain, liver weight, epididymal and perirenal fat mass, lowering the content of TG in serum and liver, and improving hepatic steatosis as well as liver redox state. However, the effects varied in different teas. Enshi Yulu Tea and Fenghuang Narcissus Tea significantly reduced body weight, and Yihong Tea was the most effective tea in preventing the elevation of epididymal and perirenal fat mass. Moreover, tea treatments might exert hypolipidemic effects by significantly decreasing serum TG level, which was particularly obvious in Enshi Yulu Tea and Fried Green Tea. More importantly, Qing Brick Tea could protect against NAFLD by evidently alleviating the increase of liver TG content, which was confirmed by the results of the liver histopathological analysis. Furthermore, Fenghuang Narcissus Tea, Enshi Yulu Tea, and Qing Brick Tea might have potential in vivo antioxidant activity. However, the increased contents of AST as well as ALT in some tea‐treatment groups indicated that some teas at a dosage of 200 mg/kg b.w. might cause liver injuries, such as Gongmei White Tea and Pu‐erh Tea. The correlation analysis results indicated that ellagic acid and gallic acid contributed to the decrease of liver weight and MDA level, respectively, and thus prevented the development of NAFLD to some extent. To sum up, these findings indicated that some teas had the potential to be developed into functional food for preventing and managing NAFLD, and the results from this study could provide a reference for the public to select teas.

## AUTHOR CONTRIBUTIONS

Conceptualization, Q.‐Q.M., R.‐Y.G. and H.‐B.L.; methodology, Q.‐Q.M., B.‐Y.L. and H.‐B.L.; software, Q.‐Q.M.; validation, Q.‐Q.M. and B.‐Y.L.; formal analysis, Q.‐Q.M. and X.‐Y.X.; investigation, Q.‐Q.M., B.‐Y.L., J.‐M.M., X.‐Y.X., Y.‐Y.G. and X.‐H.W.; resources, Q.‐Q.M.; data curation, Q.‐Q.M., B.‐Y.L. and J.‐M.M.; writing—original draft preparation, Q.‐Q.M., writing–review and editing, R.‐Y.G. and H.‐B.L.; visualization, Q.‐Q.M.; supervision, R.‐Y.G. and H.‐B.L.; project administration, H.‐B.L.; funding acquisition, R.‐Y.G. and H.‐B.L.

## ETHICAL APPROVAL

The authors declare that they do not have any conflict of interest. The study's protocols and procedures were ethically reviewed and approved by the Animal Care Committee at the School of Public Health, Sun Yat‐Sen University.

## Data Availability

The research data are not shared.
